# Perinatal Outcomes After Statin Exposure During Pregnancy

**DOI:** 10.1001/jamanetworkopen.2021.41321

**Published:** 2021-12-30

**Authors:** Jui-Chun Chang, Yen-Ju Chen, I-Chieh Chen, Wei-Szu Lin, Yi-Ming Chen, Ching-Heng Lin

**Affiliations:** 1 Department of Obstetrics and Gynecology and Women’s Health, Taichung Veterans General Hospital, Taichung, Taiwan; 2 Department of Medical Research, Taichung Veterans General Hospital, Taichung, Taiwan; 3 Division of Allergy, Immunology and Rheumatology, Department of Internal Medicine, Taichung Veterans General Hospital, Taichung, Taiwan; 4 Institute of Clinical Medicine, National Yang Ming Chiao Tung University, Taipei, Taiwan; 5 School of Medicine, National Yang Ming Chiao Tung University, Taipei, Taiwan; 6 Rong Hsing Research Center for Translational Medicine, National Chung Hsing University, Taichung, Taiwan; 7 College of Medicine, National Chung Hsing University, Taichung, Taiwan; 8 Department of Health Care Management, National Taipei University of Nursing and Health Sciences, Taipei, Taiwan; 9 Department of Industrial Engineering and Enterprise Information, Tunghai University, Taichung, Taiwan; 10 Department of Public Health, College of Medicine, Fu Jen Catholic University, New Taipei City, Taiwan; 11 Institute of Public Health and Community Medicine Research Center, National Yang Ming Chiao Tung University, Taipei, Taiwan; 12 Department of Medical Research, China Medical University Hospital, Taichung, Taiwan

## Abstract

**Importance:**

Statins are the drug class most commonly used to treat hyperlipidemia. Recently, they have been used during pregnancy for the prevention or treatment of preeclampsia. However, the safety of statin use during pregnancy has been questioned, and the sample sizes of most previous studies have been small.

**Objective:**

To examine the perinatal outcomes among offspring associated with maternal use of statins during pregnancy.

**Design, Setting, and Participants:**

This retrospective cohort study included 1 443 657 pregnant women 18 years of age or older with their first infant born during the period from January 1, 2004, to December 31, 2014. Data for this study were taken from the Taiwan National Health Insurance Research Database. Statistical analysis was performed from April 7, 2020, to July 31, 2021.

**Exposures:**

Maternal statin use during pregnancy.

**Main Outcomes and Measures:**

Women who have received a diagnosis of hyperlipidemia before pregnancy and who were receiving prescription statins during pregnancy were the statin-exposed group. Data on congenital anomalies, birth weight, gestational age, preterm birth, low birth weight, very low birth weight, fetal distress, and Apgar score were compared between participants with and partcipants without statin exposure during pregnancy. Risk ratios (RRs) were calculated by multivariable analyses using Poisson regression models to adjust for potential confounders. Subgroup analysis was performed to compare offspring of women who used statins for more than 3 months prior to pregnancy and maintained or stopped statin use after pregnancy.

**Results:**

A total of 469 women (mean [SD] age, 32.6 [5.4] years; mean [SD] gestational age, 38.4 [1.6] weeks) who used statins during pregnancy and 4690 age-matched controls (mean [SD] age, 32.0 [4.9] years; mean [SD] gestational age, 37.3 [2.4] weeks) with no statin exposure during pregnancy were enrolled. After controlling for maternal comorbidities and age, low birth weight was more common among offspring in the statin-exposed group (RR, 1.51 [95% CI, 1.05-2.16]), with a greater chance of preterm birth (RR, 1.99 [95% CI, 1.46-2.71]), and a lower 1-minute Apgar score (RR, 1.83 [95% CI, 1.04-3.20]). Congenital anomalies were not associated with statin exposure during pregnancy. In addition, multivariable analysis showed that there was no association between statin use for periconceptual hyperlipidemia and adverse perinatal outcomes among women who had used statins prior to pregnancy.

**Conclusions and Relevance:**

This study suggests that statins may be safe when used during pregnancy because there was no association with congenital anomalies, but caution is needed because of an increased risk of low birth weight and preterm labor. The data also suggest that statins could be safely used during pregnancy for women with long-term use of statins before pregnancy.

## Key Points

**Question:**

Is statin use during pregnancy associated with adverse perinatal outcomes?

**Findings:**

In this cohort study of 1 443 657 pregnancies, in which 469 women were dispensed prescription statins during pregnancy, maternal statin exposure during pregnancy was not associated with increased risk of congenital anomalies among offspring but was associated with low birth weight (<2500 g) and preterm birth compared with no exposure. There were no increases in adverse perinatal outcomes among offspring of women who took statins before conception for more than 3 months and with continuous use of statins after pregnancy compared with women who stopped statin use.

**Meaning:**

This study suggests that, although statin exposure during pregnancy was associated with preterm labor and low birth weight, there was no association between statin use for periconceptual hyperlipidemia and adverse perinatal outcomes.

## Teaser

This cohort study uses data from the Taiwan National Health Insurance Research Database to examine perinatal outcomes among offspring associated with maternal use of statins during pregnancy.

## Introduction

Statins are the class of drug most commonly used to treat hyperlipidemia and have been used during pregnancy to prevent or treat preeclampsia (PE)^1,2^ owing to their ability to reverse an angiogenic imbalance and correct endothelial dysfunction.^3^ Other benefits associated with statins are control of gestational dyslipidemia from accelerated atherosclerotic disease in the child,^4^ prevention of umbilical vein endothelial dysfunction,^5^ and spontaneous preterm birth (PTB).^6^ However, the safety of statins with regard to the fetus is of concern. The first statin drug developed, lovastatin, was designated category X (contraindicated) during pregnancy because of its possible association with congenital anomalies. The designation was based on animal studies showing developmental toxic effects and the recognition that cholesterol biosynthesis is critical to prenatal development.^7^ In the 1980s and 1990s, animal studies of statins demonstrated skeletal malformations, gastroschisis, and a low birth weight (LBW) in rats and rabbits.^8,9^ A case report of a baby born with VACTERL (vertebral, anal, cardiac, tracheal, esophageal, renal, and limb abnormalities) after in utero exposure to lovastatin has also raised concerns.^10^ Owing to ethical concerns, no large randomized clinical trial (RCT) of statin use during pregnancy has been performed, and only a few human cohort studies, case series, and 1 small RCT have focused on the use of statins during pregnancy. In most of the prior studies, the study population was small, and confounders (such as maternal comorbidities) were not fully documented.

Unnecessary or inappropriate drug use during pregnancy should be avoided, especially use of teratogenic drugs, which can irreversibly modify the growth, structure, or function of the developing embryo or fetus, resulting in potential spontaneous abortion, premature delivery, and mental or physical disabilities.^11^ The purpose of this study is to evaluate the association of statin use during pregnancy with congenital anomalies or other neonatal complications in a large national sample. Owing to ethical issues, large RCTs are difficult to perform; hence, we performed a nationwide cohort study reflecting real-world data with a large cohort.

## Methods

### Data Source

This retrospective matched cohort study was conducted using the Taiwan National Health Insurance Research Database (NHIRD), maintained by the National Health Insurance program in Taiwan that has operated since March 1, 1995, and has enrolled 99.9% of Taiwan’s population of 23.7 million individuals. The study protocol was conducted in accordance with the Declaration of Helsinki^12^ and was approved by the ethics committee of Taichung Veterans General Hospital’s institutional review board. All available data were taken from NHIRD, which is established by Taiwan’s Ministry of Health and Welfare and collected information in a standardized procedure to fit the needs of researchers’ in different fields. To protect the privacy of patients, the National Health Research Institutes has encrypted the names of patients, health care professionals, and medical institutions with unique and anonymous identifiers. Because of the anonymity of the data, informed consent was not required. This study followed the Strengthening the Reporting of Observational Studies in Epidemiology (STROBE) reporting guidelines.

### Study Design and Study Population

**Figure 1.**
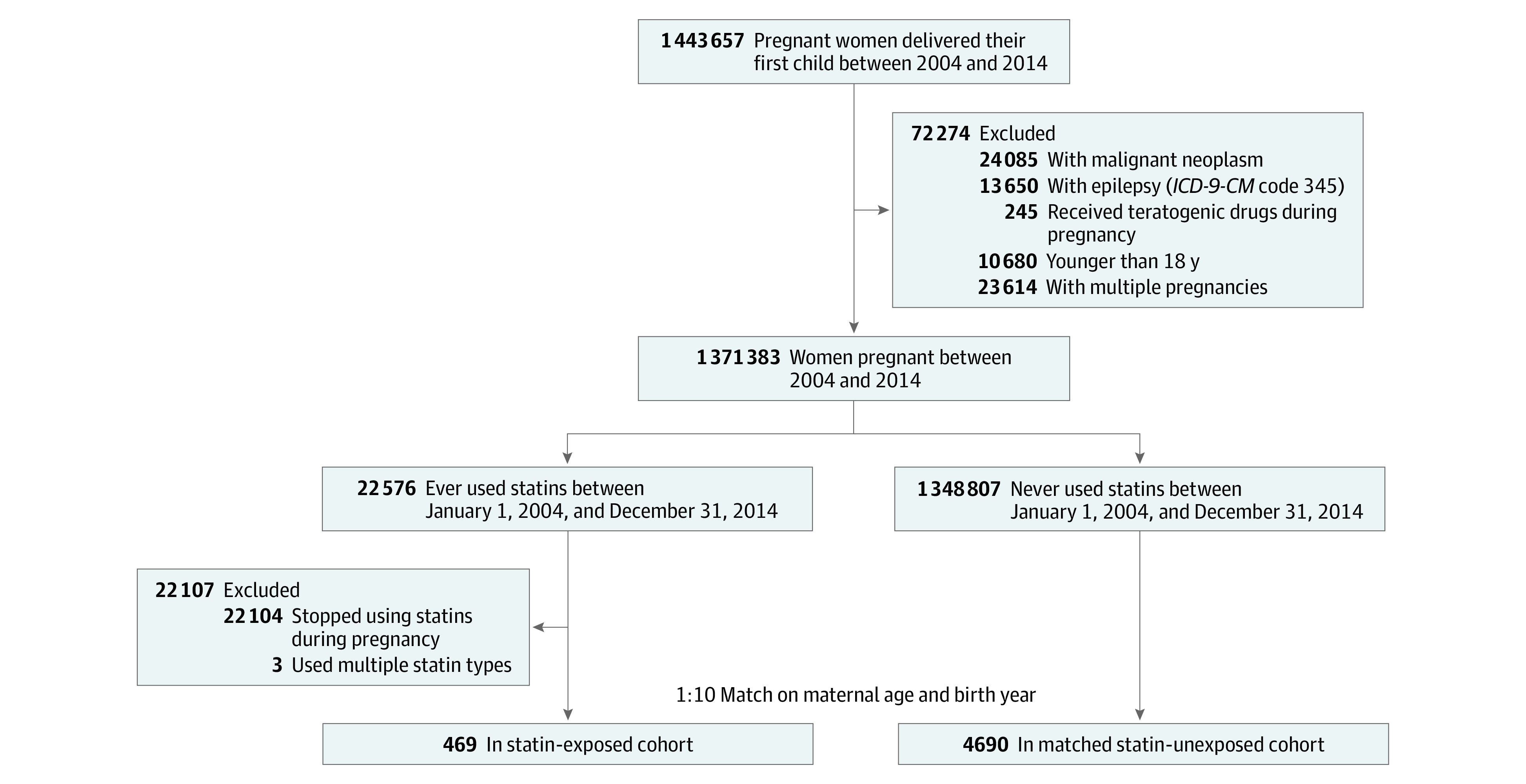
Flowchart of Participant Recruitment *ICD-9-CM* indicates International Classification of Diseases, Ninth Revision, Clinical Modification.

As shown in Figure 1, we selected women who gave birth to their first child between January 1, 2004, and December 31, 2014, as the study participants (n = 1 443 657). We excluded participants younger than 18 years of age, with multiple pregnancies, with epilepsy, and who used teratogenic drugs during pregnancy. The final study population included 1 371 356 parturient women, 22 576 of whom ever used statins during this period. Of these women, 22 104 were excluded because they were not exposed to statins during pregnancy, and 3 were excluded for exposure to both lipophilic and hydrophilic statins. Thus, there were 469 women who used prescription statins during pregnancy. The statin-exposed cohort, categorized as women who used statins during pregnancy, was derived from the NHIRD by searching for pregnant women who were prescribed statins. According to Taiwan Food and Drug Administration guidelines,^13^ statins were prescribed as indicated for dyslipidemia. Therefore, all women in the statin-exposed cohort had received a diagnosis of hyperlipidemia. The statin-unexposed cohort (n = 4690) was matched to the exposed cohort according to maternal age and year of delivery at a ratio of 1 to 10.

### Exposure

Statin use was measured by a prescription being filled for at least 7 days of use. Statins were further divided into lipophilic (lovastatin, simvastatin, fluvastatin, atorvastatin, and pitavastatin) and hydrophilic (pravastatin and rosuvastatin) categories according to lipophilicity.^14^ The statins used by the most women were atorvastatin, 10 to 40 mg daily (132 [28.1%]); rosuvastatin, 10 mg daily (82 [17.5%]); lovastatin, 50 mg daily (49 [10.4%]); simvastatin, 20 to 40 mg daily (31 [6.6%]); fluvastatin, 40 to 80 mg daily (25 [5.3%]); and pravastatin. 40 mg daily (8 [1.7%]); all other statins combined were used by 30.3% of the participants (142).

### Covariates

Baseline characteristics, including maternal age and comorbidities prior to pregnancy, were analyzed, and hypertension (*International Statistical Classification of Diseases and Related Health Problems, Tenth Revision, Clinical Modification* [*ICD-10-CM*] codes 401-405) and diabetes (*International Classification of Diseases, Ninth Revision, Clinical Modification* [*ICD-9-CM*] code 250) were recorded. Hypertension and diabetes were diagnosed before pregnancy. Diabetes was diagnosed based on a random blood glucose level of 200 mg/dL or more (to convert glucose to millimoles per liter, multiply by 0.0555) or a fasting blood glucose level of 126 mg/dL or more, or a hemoglobin A_1c_ level of 6.5% or more (to convert to proportion of total hemoglobin, multiply by 0.01). Hypertension was diagnosed as blood pressure at 2 to 3 office visits of 140/90 mm Hg or more. The primary study outcome, congenital malformations, was defined based on the diagnosis of 1 or more organ-specific malformations (*ICD-9-CM* code 740-759). Secondary outcomes included birth weight, gestational age, PTB (gestational age, <37 weeks), LBW (<2500 g), very LBW (<1500 g), fetal distress (*ICD-9-CM* code 656.3), and Apgar score at 1 minute and 5 minutes.

**Figure 2.**
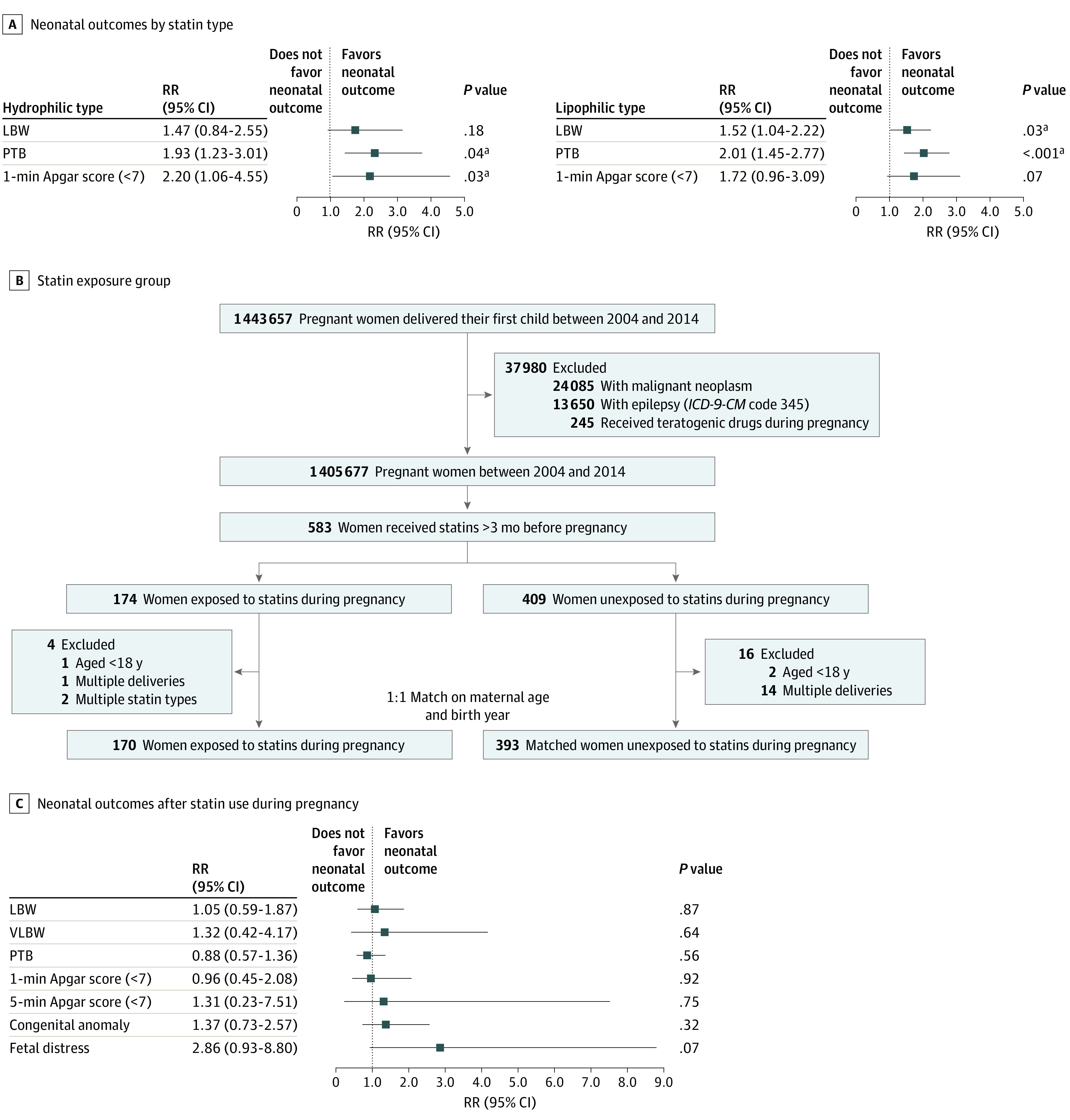
Multivariable Analysis of Factors Associated With Neonatal Outcomes A, Adjusted risk ratios (RRs) of neonatal outcomes among women using different types of statins during pregnancy. B, Flowchart of the analysis of the preconception statin-exposed group. C, Adjusted RRs of neonatal outcomes among women using statins for more than 3 months prior to pregnancy and maintaining use during pregnancy. Adjusted RRs were obtained after stratification for maternal age, pregestational hypertension, and diabetes. Risk ratios for individual studies are indicated by squares and 95% CIs by horizontal lines. LBW indicates low birth weight; PTB, preterm birth; and VLBW, very low birth weight. ^a^Statistically significant difference at *P* < .05.

We then compared the neonatal outcomes specifically for women who had used statins prior to pregnancy. The subgroup with preconception statin exposure was defined as women who had used statins for more than 3 months prior to pregnancy. A total of 563 women used statins for more than 3 months prior to pregnancy; 170 continued using statins after conception, and 393 stopped using statins after conception. The 2 groups were matched by maternal age and delivery year in a ratio of 1 to 1 (Figure 2B).

### Statistical Analysis

Statistical analysis was performed from April 7, 2020, to July 31, 2021. For demographic data, continuous variables are shown as the mean (SD) values, and categorical variables are shown as number (percentage). The χ^2^ test and the *t* test were used to compare variables between the statin-exposed and statin-unexposed cohorts. The risk ratio (RR) for congenital anomalies, PTB, LBW, very LBW, Apgar score at 1 minute and 5 minutes, and fetal distress associated with statin use was estimated by a Poisson regression model with robust error variance. For exploratory purposes, regressions were adjusted for potential confounders. All data were analyzed using SAS, version 9.4 software (SAS Institute Inc). All *P* values were from 2-sided tests and results were deemed statistically significant at *P* < .05.

## Results

We identified 469 pregnant women who were 18 years or older with data recorded in the electronic health record. Categories of maternal age and maternal comorbidity are presented in Table 1. A total of 469 primiparous mothers (mean [SD] age, 32.6 [5.4] years) who used statins during pregnancy and 4690 age-matched controls (mean [SD] age, 32.0 [4.9] years) with no statin exposure during pregnancy were enrolled. The prevalence of diabetes (196 [41.8%] vs 20 [0.4%]; *P* < .001) and hypertension (121 [25.8%] vs 53 [1.1%]; *P* < .001) were significantly higher in the statin-exposed cohort. The mean (SD) gestational age was 38.4 (1.6) weeks in the statin-exposed cohort and 37.3 (2.4) weeks in the statin-unexposed cohort. The mean (SD) birth weight was 3105.62 (444.3) g for infants in the statin-exposed cohort and 3035 (684.1) g for infants in the statin-unexposed cohort.

**Table 1.** Characteristics of Study Population

Maternal characteristic	Cohort, No. (%)		Total No.	*P* value^a^
	Statin exposed (n = 469)	Statin unexposed (n = 4690)		
Age, y				
18-29	116 (24.7)	1160 (24.7)	1276	>.99
30-34	180 (38.4)	1800 (38.4)	1980	
≥35	173 (36.9)	1730 (36.9)	1903	
Comorbidity				
Diabetes	196 (41.8)	20 (0.4)	216	<.001
Hypertension	121 (25.8)	53 (1.1)	174	<.001

^a^ The χ^2^ test and the *t* test were used to compare variables between the statin-exposed and statin-unexposed cohorts.

Multivariable analysis showed that, compared with mothers in the statin-unexposed group, statin-exposed mothers had significantly greater risks of developing PE or eclampsia (RR, 2.78 [95% CI, 1.66-4.65]), and their offspring had significantly greater risks of PTB (RR, 1.99 [95% CI, 1.46-2.71]), LBW (RR, 1.51 [95% CI, 1.05-2.16]), and having a lower 1-minute Apgar score (RR, 1.83 [95% CI, 1.04-3.20]) (Table 2). After adjustment for maternal age and comorbidities, the multivariable analysis of factors associated with congenital anomalies showed that the congenital anomaly was no longer associated with statin exposure (RR, 1.24 [95% CI, 0.81-1.90]), and it was only associated with pregestational diabetes (RR, 2.29 [95% CI, 1.38-3.80]) after adjusting for maternal age and comorbidities (Table 3). For women without diabetes or hypertension, statin exposure was associated with PTB (RR, 1.88 [95% CI, 1.28-2.75]) (eTable 1 in the Supplement). When statins were categorized into hydrophilic and lipophilic types, multivariable analysis showed that both types were associated with PTB (hydrophilic: RR, 1.93 [95% CI, 1.23-3.01]; lipophilic: RR, 2.01 [95% CI, 1.45-2.77]); only the lipophilic type was associated with LBW (RR, 1.52 [95% CI, 1.04-2.22]), and only the hydrophilic type was associated with a low 1-minute Apgar score (RR, 2.20 [95% CI, 1.06-4.55]) (Figure 2A).

**Table 2.** Clinical Characteristics of Study Population

Characteristic	Cohort, No. (%)		RR (95% CI)^a^
	Statin exposed (n = 469)	Statin unexposed (n = 4690)	
Neonatal outcome			
Preterm birth (<37 wk)	118 (25.2)	343 (7.3)	1.99 (1.46-2.71)^b^
Low birth weight (<2500 g)	72 (15.4)	312 (6.7)	1.51 (1.05-2.16)^c^
Very low birth weight (<1500 g)	17 (3.6)	24 (0.5)	2.41 (0.90-6.44)
Apgar score (<7)			
At 1 min	44 (9.4)	105 (2.2)	1.83 (1.04-3.20)^c^
At 5 min	9 (1.9)	21 (0.4)	0.96 (0.24-3.90)
Congenital anomaly	55 (11.7)	289 (6.2)	1.24 (0.81-1.90)
Fetal distress	31 (6.6)	158 (3.4)	1.01 (0.55-1.86)
Maternal outcome			
Gestational			
Diabetes	18 (3.8)	123 (2.6)	1.07 (0.60-1.92)
Hypertension	12 (2.6)	34 (0.7)	1.90 (0.68-5.33)
Preeclampsia or eclampsia	66 (14.1)	77 (1.6)	2.78 (1.66-4.65)^b^
Placenta previa and abruptio placentae	23 (4.9)	190 (4.1)	1.17 (0.67-2.04)
Cesarean delivery	266 (56.7)	1840 (39.2)	1.17 (0.98-1.40)

Abbreviation: RR, risk ratio.

^a^ The Poisson regression model was used to compare variables between the statin-exposed and statin-unexposed cohorts and adjusted for potential confounders.

^b^ *P* < .001.

^c^ *P* < .05.

**Table 3.** Multivariable Analysis of Factors Associated With Congenital Anomaly

Maternal characteristic	Congenital anomaly, RR (95% CI)^a^
Statin-exposed women	1.24 (0.81-1.90)
Age, y	
18-29	1 [Reference]
30-34	0.87 (0.66-1.14)
≥35	0.93 (0.71-1.21)
Comorbidity	
Diabetes	2.29 (1.38-3.80)
Hypertension	1.00 (0.60-1.68)

Abbreviation: RR, risk ratio.

^a^ The Poisson regression model was used to compare variables between the statin-exposed and statin-unexposed cohorts and adjusted for potential confounders.

In preconception analysis of the statin-exposure group, the occurrences of pregestational diabetes (98 of 170 [57.6%] vs 68 of 170 [40.0%]; *P* = .001) and hypertension (63 of 170 [37.1%] vs 46 of 170 [27.1%]; *P* = .05) were higher in the group with continuous statin exposure. Multivariable analysis showed the prenatal outcomes to be comparable between groups, including congenital anomalies (RR, 1.37; 95% CI, 0.73-2.57), LBW (RR, 1.05; 95% CI, 0.59-1.87), very LBW (RR, 1.32; 95% CI, 0.42-4.17), PTB (RR, 0.88; 95% CI, 0.57-1.36), 1-minute Apgar score (RR, 0.96; 95% CI, 0.45-2.08), 5-minute Apgar score (RR, 1.31; 95% CI, 0.23-7.51), and fetal distress (RR, 2.86; 95% CI, 0.93-8.80) (Figure 2C). These results indicated that statin exposure during pregnancy was associated with preterm labor and LBW, but there was no association between statin use for periconceptual hyperlipidemia and adverse perinatal outcomes.

## Discussion

In this cohort study, the women who used statins had a higher prevalence of comorbid conditions. After adjustment for confounding factors, statin exposure during pregnancy was not associated with congenital anomalies in offspring but was associated with LBW, PTB, and a low 1-minute Apgar score. For women who used statins prior to pregnancy for more than 3 months, maintaining statin use during pregnancy did not increase the risk of adverse neonatal outcomes, including congenital anomalies, LBW, PTB, very LBW, low Apgar scores, and fetal distress.

Statins are contraindicated during pregnancy owing to possible teratogenicity, which may be caused by the interruption of cholesterol synthetization. Cholesterol and its derivatives are considered essential components for fetal development, and they are involved in the synthesis of steroids and cell membranes. Cholesterol synthesis is crucial for the development of both the central and peripheral nervous systems.^15,16,17^ In rats, inhibition of 3-hydroxy-3-methylglutaryl coenzyme A reductase by simvastatin was found to significantly reduce fetal testosterone production.^18^ However, to our knowledge, few studies have explored the association of maternal cholesterol reduction with neonatal outcomes, despite concerns regarding teratogenicity arising from animal models.^19^ The results of our study showed that statin exposure during pregnancy was not associated with congenital anomalies. This finding is inconsistent with previous animal studies and human reports that showed central nervous system and limb anomalies in offspring of mothers with statin exposure during pregnancy.^10,20^ However, the results were comparable to those of recent studies. The largest cohort study, performed by Bateman et al,^21^ reviewed the records of 888 996 completed pregnancies linked to live births of women enrolled in Medicaid and identified a cohort of 1152 women (0.1%) who had filled a prescription for a statin during the first trimester of their pregnancy. In that study, the statin users had a higher prevalence of comorbidity, but after adjustment for confounding variables, statin exposure was not associated with an increased risk of congenital anomaly. However, the study did not explore other neonatal outcomes. One systematic review also concluded that there was no clear association of congenital anomalies with statin use during pregnancy, and the findings indicated that statins are probably not teratogenic in humans.^19^

The results of our study showed that offspring of women in the statin-exposed group tended to have LBW and an earlier gestational age; we also found that statin exposure was associated with LBW and greater risk of PTB. To clarify the results, we also analyzed our data to see whether statin exposure was associated with small for gestational age status, defined as birth weight below the 10th percentile for gestational age according to the Fenton preterm growth chart, which is used in many countries (eTable 2 in the Supplement).^22^ The result showed that statin exposure is not associated with small for gestational age status after adjusting for maternal age and the comorbidities of hypertension and diabetes (RR, 0.99; 95% CI, 0.73-1.35). Few studies have discussed other neonatal outcomes, with the exception of congenital anomalies, and, to our knowledge, no study has focused on the rate of LBW. One cohort study comparing 64 women receiving statins with 64 controls of a similar age revealed that the mean (SD) gestational age at birth (38.4 [2.8] weeks vs 39.3 [1.3] weeks; *P* = .04) and birth weight (3.14 [0.68] kg vs 3.45 [0.42] kg; *P* = .01) were lower in the statin-exposed group.^23^ That study did not focus on the rates of PTB and LBW, and the results were not stratified by type of statin. Another cohort study of 249 statin-exposed pregnancies and 249 controls concluded that PTB was more frequent during statin-exposed pregnancies (16.1% vs 8.5%; odds ratio, 2.1; 95% CI, 1.1-3.8), but the median gestational age at birth and birth weight did not differ between the groups.^24^ Other studies of pravastatin, a hydrophilic-type statin used for the prevention or treatment of PE, did not find that statin exposure during pregnancy was associated with a greater risk of LBW.^25,26^

In our study, a low 1-minute Apgar score (<7) was seen in the statin-exposed group, but there was no significant difference in the 5-minute Apgar score. Many factors can influence the Apgar score, including maternal sedation or anesthesia, congenital malformations, gestational age, trauma, and interobserver variability.^27^ The lower 1-minute Apgar score may be a sign that these infants may more frequently require resuscitation after birth.

We further analyzed the different types of statins and found that the lipophilic type was associated with LBW. It has been suggested that hydrophilic statins, such as pravastatin, are less likely to enter the embryo during pregnancy. They have less potential to affect cholesterol biosynthesis and are less likely to adversely affect the developing infant.^28^ The results of our study also confirmed this finding, as the hydrophilic type was associated with more adverse outcomes. A systematic review identified 3 major congenital anomalies and 1 minor congenital anomaly in 138 infants exposed to pravastatin, and it concluded that the available information does not allow one to draw any conclusions regarding the risk of adverse outcomes during pregnancy based on whether a statin is lipophilic or hydrophilic.^19^ Recent RCTs on pravastatin use during pregnancy for the prevention or treatment of PE also showed no identifiable safety risks associated with pravastatin use in their cohorts.^25,29^

In terms of maternal outcomes, more women in the statin-exposed group developed PE. This finding is compatible with a previous study’s finding that maternal preconception dyslipidemia is associated with PE.^30^ There are an increasing number of studies evaluating the association of statin use with treatment or prevention of PE owing to statins’ properties and mechanisms of action.^3,25,29^ However, the results are inconsistent. We did not see a protective effect of statins in our study. One recent RCT also showed that women with early-onset PE (24 weeks and 0 days to 31 weeks and 6 days of gestation) who received pravastatin, 40 mg daily, had a similar length of pregnancy compared with those who received placebo, and the plasma soluble fms-like tyrosine kinase 1 levels were not lower in the pravastatin group.^29^

In a subgroup analysis, we focused on women who had used statins for more than 3 months prior to pregnancy. This group included women with hyperlipidemia and a greater risk of developing PE or gestational diabetes during pregnancy, which may result in poorer neonatal outcomes.^30^ No increased risks of adverse neonatal outcomes were identified in the group who continuously used statins compared with the group who stopped using statins after conception. According to the American College of Cardiology/American Heart Association guidelines, women of childbearing age with a level of low-density lipoprotein cholesterol greater than 190 mg/dL (to convert to millimoles per liter, multiply by 0.0259) or those with diabetes who were older than 40 years of age should receive statin treatment to reduce the risk of atherosclerotic cardiovascular disease.^31^ To our knowledge, no studies have evaluated the long-term cardiovascular outcomes of cessation of treatment among women taking statins who discontinue use prior to or during pregnancy.^1^ However, there is strong evidence to indicate that discontinuation of statin therapy increases incidences of cardiovascular and cerebrovascular events.^32,33^ In addition, studies have shown that children born to mothers with a higher blood cholesterol level during pregnancy are more likely to have advanced aortic atherosclerotic plaques.^4,34^ The necessity of statins for the treatment of dyslipidemia during pregnancy should be evaluated.

### Research Implications

Our findings suggested that statins may be used during pregnancy with no increase in the rate of congenital anomalies. For pregnant women at low risk, statins should be used carefully after assessing the risks of LBW and PTB. For women with dyslipidemia or high-risk cardiovascular disease, as well as those who use statins before conception, statins may be continuously used with no increased risks of neonatal adverse effects.

### Strengths and Limitations

Our study had several strengths. First, up to 99.99% of Taiwan’s population are enrolled under the National Health Insurance program,^35^ which provides a population-level data source for the generation of real-world evidence to support clinical decisions. Moreover, information on statin use within the database was derived from issued prescriptions rather than self-report, so our findings were not susceptible to recall bias. According to the birth reporting system in Taiwan, the rate of newborns delivered in hospitals or clinics is up to 99.8%^36^; information regarding newborn and maternal status can thus be taken to be recorded precisely. Therefore, we were able to perform the largest cohort analysis to date, to our knowledge, examining other neonatal outcomes in addition to congenital anomalies that are seldom mentioned in previous studies.

Our study also had some limitations. First, this was a cohort study, not an RCT. However, it could reflect real-world data without selection bias, and owing to the uncertainty surrounding the benefits associated with statin use during pregnancy, large RCTs are difficult to perform. Second, maternal conditions (such as smoking status, body mass index, and previous birth histories), which may be associated with neonatal outcomes, were not recorded. According to the report of the Adult Smoking Behavior Surveillance System, 2.3% to 5.1% of women aged 18 years or older in Taiwan during the period from 2004 to 2014 were smokers, and half of the women who did smoke quit when they were pregnant.^37^ Owing to the low rate, smoking status may not have been associated with our results. Third, the subgroup with preconception statin exposure was relatively small because statin use due to dyslipidemia was less frequent among women of reproductive age and because women preparing for pregnancy would stop statin use because of concerns about safety in pregnancy. Another limitation was that the database includes information only on live births, and the study outcomes cannot be applied to cases of miscarriage or intrauterine fetal death. A prospective study may be conducted to evaluate the correlations.

## Conclusions

This nationwide cohort study suggests that statins may be used safely during pregnancy because they were not associated with congenital anomalies but need to be used with caution owing to increased risk of LBW and PTB. Further work must be performed to confirm our findings.

## Notes

Supplement. **eTable 1.** Multivariable Analysis of Diabetes Mellitus or Hypertension Associated With Preterm Birth **eTable 2.** Multivariable Analysis of Factors Associated With Small for Gestational Age
