# Supplementary material for: Perinatal Outcomes After Statin Exposure During Pregnancy
Source: JAMA Netw Open. 2021 Dec 30;4(12):e2141321. doi: 10.1001/jamanetworkopen.2021.41321 (PMC8719244; doi:10.1001/jamanetworkopen.2021.41321)
Supplement: Supplement. — eTable 1. Multivariable Analysis of Diabetes Mellitus or Hypertension Associated With Preterm Birth eTable 2. Multivariable Analysis of Factors Associated With Small for Gestational Age [file jamanetwopen-e2141321-s001.pdf]

Supplementary Online Content

Chang JC, Chen YJ, Chen IC, Lin WS, Chen YM, Lin CH. Perinatal outcomes after statin exposure during pregnancy. *JAMA Netw Open.* 2021;4(12):e2141321. doi:10.1001/jamanetworkopen.2021.41321

eTable 1. Multivariable Analysis of Diabetes Mellitus or Hypertension Associated With Preterm Birth
eTable 2. Multivariable Analysis of Factors Associated With Small for Gestational Age

This supplementarymaterial has been provided by the authors to givereadersadditional information about theirwork.

© 2021 Chang JC et al. *JAMA Network Open.*

eTable 1. Multivariable Analysis of Diabetes Mellitus or Hypertension Associated With Preterm Birth

**Characteristic**	**Preterm Birth (<37 weeks)**	
	**Diabetes mellitus/ Hypertension (0/0)**	
	**RR (95% CI)**	***P*value[a]**
**Statin-exposed women**	1.88 (1.28-2.75)	.001
**Maternal age (years)**		
18-29	1 [Reference]	
30-34	1.04 (0.79-1.37)	.80
≥35	1.23 (0.94-1.62)	.14

[a]The Poisson regression model was used to compare variables between the statin-exposed and -unexposed cohorts.

© 2021 Chang JC et al. *JAMA Network Open.*

eTable2. Multivariable Analysis of Factors Associated With Small for Gestational Age

Characteristic	Low Birth Weight							
	All		Week <34		Week 34-36+6d		Week≥37	
	RR (95% CI)	*P*value[a]	RR (95% CI)	*P*value[a]	RR (95% CI)	*P*value[a]	RR (95% CI)	*P*value[a]
**Statin-exposed women**	0.99 (0.73-1.35)	.97	1.13 (0.20-6.42)	.89	0.97 (0.34-2.79)	.95	1.02 (0.74-1.42)	.88
**Maternal age (years)**								
18-29	1 [Reference]		1 [Reference]		1 [Reference]		1 [Reference]	
30-34	0.80 (0.67-0.95)	.01	0.80 (0.05-13.75)	.88	1.56 (0.61-4.04)	.36	0.78 (0.66-0.93)	.007
≥35	0.77 (0.65-0.92)	.004	2.72 (0.31-23.63)	.37	1.23 (0.47-3.25)	.67	0.76 (0.63-0.91)	.003
**Maternal comorbidity**								
Diabetes mellitus	0.66 (0.41-1.06)	.09	0.98 (0.17-5.69)	.99	0.38 (0.10-1.55)	.18	0.77 (0.45-1.31)	.33
Hypertension	1.61 (1.10-2.37)	.01	3.12 (0.61-15.86)	.17	2.87 (1.12-7.35)	.03	1.50 (0.93-2.40)	.09

[a] Adjusting maternal age and comorbidity, hypertension and DM.

© 2021 Chang JC et al. *JAMA Network Open.*
